# Coronal and sagittal balance in Lenke 5 AIS patients following posterior fusion: important role of the lowest instrument vertebrae selection

**DOI:** 10.1186/s12891-018-2135-2

**Published:** 2018-07-09

**Authors:** Xi Yang, Bowen Hu, Yueming Song, Limin Liu, Chunguang Zhou, Zhongjie Zhou, Ganjun Feng

**Affiliations:** Department of Orthopedics Surgery, West China Hospital, Sichuan University, No. 37 GuoXue Road, Chengdu, 610041 Sichuan China

**Keywords:** Adolescent idiopathic scoliosis, Thoracolumbar/lumbar curve, Posterior surgery, Coronal balance, Sagittal balance, LIV

## Abstract

**Background:**

Lenke 5 AIS is a kind of three-dimensional deformity and literature reported it usually accompany with coronal or/and sagittal imbalance. However, the postoperative coronal and sagittal balance in these patients has rarely be analyzed previously and the predict factors for postoperative trunk balance are still unclear. To synthetically analysis coronal and sagittal balance of Lenke 5 AIS patients simultaneously and found out predict factors for postoperative coronal or/and sagittal imbalance.

**Methods:**

Fifty-six Lenke 5 AIS patients who underwent posterior surgery and be followed up more than 2 years were included in this study. Coronal parameters included main curve Cobb angle, lumbosacral hemi-curve Cobb angle, preoperative LEV/LIV tilt and translation and C7-CSVL distance; While sagittal parameters included pelvic incidence(PI), sacral slope(SS), pelvic tilt(PT), lumbar lordosis(LL), thoracic kyphosis(TK), and sagittal vertical axis(SVA). Coronal imbalance was defined as C7-CSVL> 20 mm, and sagittal imbalance defined as (1) SVA > 40 mm or (2) PT < 20% PI/2 or PT > 20° or (3) PI-LL > 10°. And relative parameters were compared between balance and imbalance group to find out predict factors.

**Results:**

All seven final coronal imbalance patients occurred in LIV = L5 group. Preoperative LIV tilt(11.4°) and translation(5.2 mm) in coronal imbalance group were abnormally lower than balance group (21.7° and 15.7 mm respectively). Eighteen patients performed final sagittal imbalance. The PI in these patients (37.7°) was significantly lower than balance group (48.0°). And most of finial sagittal imbalance patients also occurred in LIV = L5 group.

**Conclusions:**

LIV = L5 as a threshold point, represents higher risk of postoperative coronal and/or sagittal imbalance. Besides, large LEV-S1 curve in reduce-bending film and small PI is directly related to final coronal imbalance and sagittal imbalance respectively.

## Background

The goal of corrective surgery in adolescent idiopathic scoliosis (AIS), is far more than straightening the coronal curve, but is meant to achieve global spinal balance and save more spine motion function. However, the scoliosis correction and spine motion level reserving in fact are opposed to each other. To obtain the best surgical outcomes, various previous studies were conducted with the same aim to find out the optimum fusion design taking into consideration the deformity, balance and function together. Lenke 5 is a particular type of AIS defined as those patents that have a structural thoracolumbar or lumbar (TL/L) scoliosis [1]. And a large portion of these patients who show coronal or sagittal spinal imbalance can be found in the literature [2, 3]. So, many recent studies turn to pay attention to the postoperative balance improvement and the corresponding influence factors [4–6].

Ideally, spinal alignment in the coronal plane is symmetrical, so coronal balance is a relatively clear concept in the AIS patients. For the patients with Lenke 5 AIS, it has been found that nearly 57% of patients show coronal imbalance before surgery [2]. And a strong correlation has been documented between the selection of the lowest instrumented vertebra (LIV) and postoperative coronal imbalance [2, 5]. In an earlier study, Li et al. found it will be a high risk of postoperative coronal imbalance when chose LIV in the vertebra that tilt more than 25° to horizon line [5]. Their conclusion was supported by a few other studies, such as Wang et al. [2] and Roberts et al. [7]. However, this viewpoint was questioned by another more recent study from Sun et al. [8]. Up to the present, it is still unclear how LIV influences postoperative coronal balance.

AIS is a kind of three-dimensional deformity. Sagittal balance is equally as important as coronal balance in terms of the overall evaluation of AIS. However, unlikely coronal alignment which is easy to understand and evaluate as the coronal plane of a normal person is upright and symmetrical, it is difficult to evaluate sagittal alignment for the lack of standardized parameters for reference due to its complex curved appearance on the lateral view. For this reason, the existing studies about sagittal balance in AIS patients are rare. In fact, sagittal balance should be understood as an optimal interaction of the spine motion segment and pelvis which eventually adjusts body gravity center above the hip joint and keeps standing posture with minimum energy cost [9, 10]. Gravity line over-shifting, pelvic-lumbar alignment mismatch, and abnormal pelvic posture are considered to be patterns of sagittal imbalance. In our previous study, we had already found that about half (48%) of the Lenke 5 AIS patients were suffering from excessively anteverted pelvic sagittal posture with an abnormally small pelvic tilt (PT) before the surgery [3]. And we found LIV selection also has a close correlation with postoperative unrecovered anteverted pelvis [3]. But, until now, the overview of the sagittal balance, as well as the specific influence of LIV in postoperative sagittal imbalance in these patients still remain unknown.

To our knowledge, there has not been any published literature which analyzed both coronal balance and sagittal balance in patients with Lenke 5 AIS in the same study. So, a series of questions remain unanswered. Is the coronal imbalance associated with sagittal imbalance? What is the specific influence of LIV selection to coronal and sagittal balance after surgery? Besides LIV, is there any other predictive factor that is related to postoperative coronal or sagittal balance? To answer these questions, we designed a retrospective study to synthetically analyze coronal and sagittal balance radiographic parameters in the same database of Lenke 5 patients.

## Methods

This was a retrospective study of 56 patients with Lenke type 5 AIS who underwent corrective posterior-only surgery in our department from Jan. 2010 to Jan. 2015. Indication for surgery was a Cobb angle of the TL/L curve > 40 degrees. All these patients had a more than 2 years follow-up after surgery with complete follow-up radiographic data. And this study was also approved by the local Ethics Committee.

### Surgical technique

All surgeries were performed by the same surgical team. The fusion area covered the structural thoracolumbar/lumbar scoliosis. The lowest level to be instrumented (LIV) was chosen according to preoperative PA and lateral side-bending films. Generally the fusion was stopped at LEV, but it may be extended below the LEV once the case meet one of the following criteria: (1) main TL/L curve cobb > 60°; (2) the disc wedging between the LEV and LEV+ 1 cannot reduce to parallel in bending film; (3) prominent vertebral tilt or translation of the LEV. Several surgical maneuvers were utilized in the operation, including rod-rotation, apical vertebral derotation (by vertebral column manipulation or vertebral coplanar alignment appliance). The Legacy or CD Horizon M8 screw-rod system (Medtronic Sofamor Danek USA, Inc. Memphis, TN) were used for fixation in these patients.

### Radiographic assessment

Standing full-length posteroanterior (PA) and lateral X-films by the multi-purpose Digital R/F System (Sonial Vision Safire 17, Shimadzu Corporation) were performed routinely before surgery and at each out-patient review after surgery. The full-length side-bending films were taken only before surgery. All radiological parameters were measured by two attending spinal surgeons, and then their average value was adopted.

Coronal parameters contains: main TL/L Cobb angle; lumbosacral hemi-curve Cobb angle (including neutral position and reduce-bending position), preoperative LEV tilt and translation, preoperative LIV tilt and translation, and C7-CSVL distance.

Lumbosacral hemi-curve was measured as the Cobb angle between inferior endplate of the LEV and the superior endplate of the S1. LEV/LIV tilt was measured as the angle between the inferior endplate of LEV/LIV to the horizon line. LEV/LIV translation was measured as the horizontal distance between the geometric center of LEV/LIV to the CSVL line. C7-CSVL distance was measured as the horizontal distance between the center of C7 plumb line to the CSVL line. If the C7 plumb line shifted to the preoperative convex side relative to the CSVL, then the C7-CSVL and LEV/LIV translation were defined as positive value, otherwise as negative value. And the coronal imbalance was defined when the absolute value of C7-CSVL distance exceeding than 20 mm [11].

Sagittal parameters contains: pelvic incidence (PI), sacral slope (SS), pelvic tilt (PT), lumbar lordosis (LL), PI-LL, thoracic kyphosis (TK), and sagittal vertical axis (SVA).

PI, SS and PT were measured as previous standard methods. LL was measured as the Cobb angle between the upper endplate of the L1 and S1. PI-LL was calculated by the value of PI minus LL value. TK was measured as the Cobb angle between the upper endplate of the T1 and L1. SVA was defined as the horizontal distance between the center of the C7 vertebral body and the posterior superior corner of the sacrum. A positive value indicated the C7 plumb line anterior to sacrum posterior corner, while negative value was the line posterior to the corner.

The present criteria of sagittal imbalance combined the common pelvic abnormal posture in Lenke 5 patients and relevant standard derived from SRS-Schwab classification [3, 12]: (1) The absolute value of SVA exceeding to 40 mm; (2) PT less than 20% of PI/2 or more than 20°; (3) The absolute value of PI-LL exceeding to 10°. To meet one or more of the above criteria will be determined as sagittal imbalance.

### Statistical analysis

All data were analyzed by SPSS 17 statistical analysis software and expressed as mean ± standard deviation (SD). Quantitative data were analyzed by T test or Mann-Whitney test as appropriate (including age, Risser sign, and all coronal or sagittal quantitative parameters). Categorical data were analyzed by χ2 test (including gender and LIV selection). Spearman correlation analysis was carried out between coronal and sagittal balance status (including postoperative, last follow-up). A value of *P* < 0.05 was considered statistically significant.

## Result

The age of 56 Lenke 5 AIS patients in this study was 15.2 ± 2.1 years. There were 36 female and 20 male with Risser sign of 3 ± 1. Main TL/L curve Cobb angle on average was 53.0 ± 8.4° before surgery and 6.8 ± 6.7° at final follow-up. The LIV selection in these patient were LIV = L3 in 9, L4 in 23, and L5 in 21 patients. The mean follow-up of these patients was 34 months (range from 24 to 72 months).

### Coronal balance

Before surgery, there were 23 in 56 (41%) patients show coronal imbalance. When comparing these patients to other ones with coronal balance (*n* = 33), no significant difference was found in age (*p* = 0.941), gender (*p* = 0.464) or Risser sign (*p* = 0.956). The main TL/L curve Cobb angle was 51.6 ± 7.4°in coronal imbalance patients and 54.0 ± 9.0° in balance ones with no significant difference (*p* = 0.301). Similarly, there was no significant difference can found between these two group patients in LEV tilt (imbalance 30.1 ± 6.0° vs. balance 28.3 ± 5.0°, *p* = 0.242), LEV translation (27.3 ± 8.3 mm vs. 26.8 ± 8.9 mm, *p* = 0.838), or Lumbosacral Hemi-curve neutral Cobb angle (25.5 ± 7.5° vs. 23.6 ± 6.4°, *p* = 0.317) or Reduce-bending Cobb angle (7.6 ± 7.8° vs. 7.4 ± 6.1°, *p* = 0.917).

**Fig. 1:**
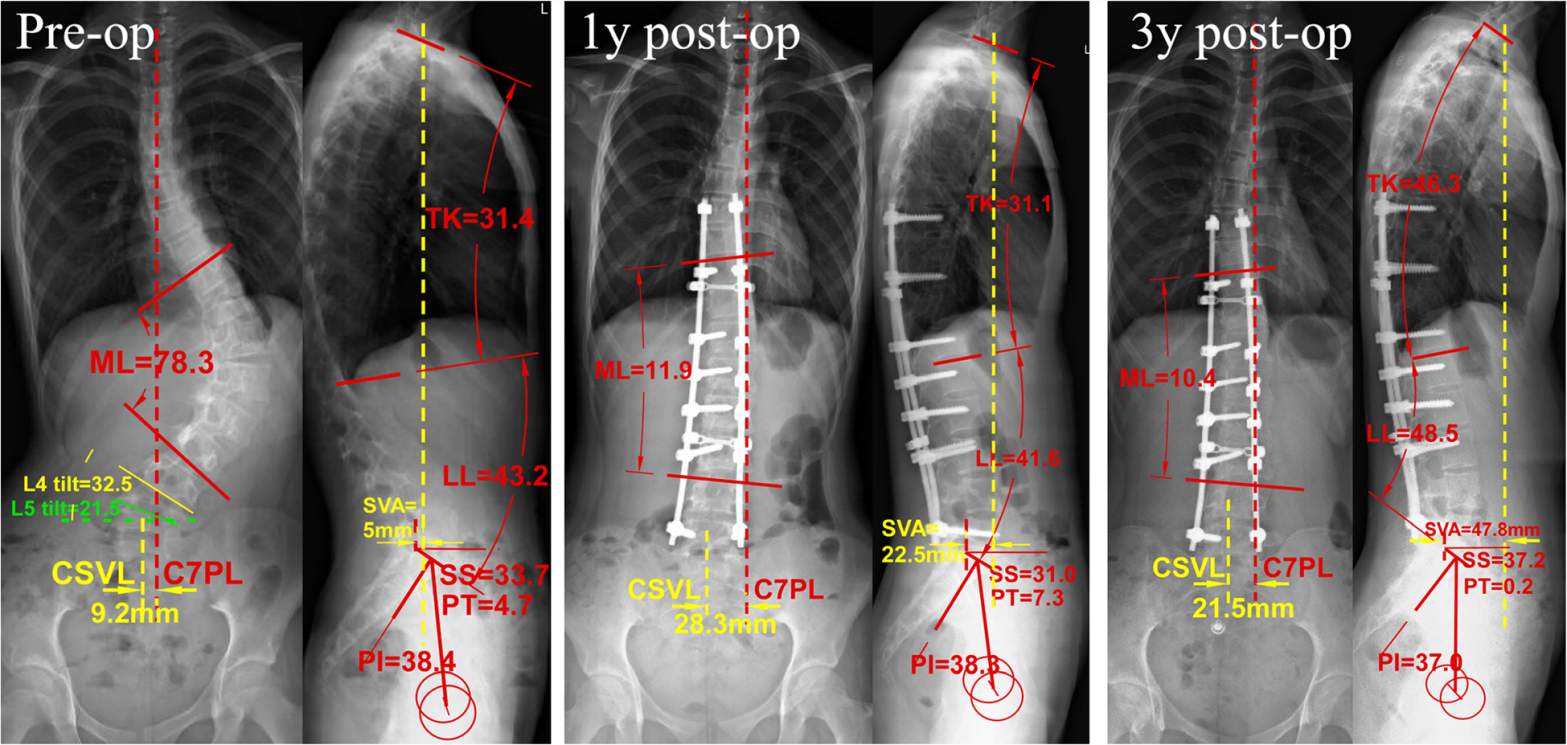
The pre-, 1-year post- and 3-year post-operative X rays of a Lenke 5 AIS patient with final coronal imbalance. Preoperatively, the main lumbar Cobb angle was 78.3° with normal coronal and sagittal balance. Both LEV (L3) tilt and LEV+ 1 (L4) tilt were exceeding to 25°, according to experience in literatures, we extended the fusion to lower level (L5 tilt = 21.5°). At 1 year after surgery, the main lumbar curve has been decreased to 11.9° with a corrective rate of 84.8%. However, the patient performed coronal imbalance with C7-CSVL distance of 28.3 mm. At last follow-up (3 years after), her coronal imbalance had a little improvement with C7-CSVL distance of 21.5 mm, still belong to coronal imbalance. And this time, this patient also performed sagittal imbalance with SVA = 47.8 mm and PT = 0.2°. The possible reasons were her smaller PI value (37°) and simultaneously been fused to L5

At the last follow-up, the coronal imbalance has still been found in 7 patients (Table 1). Four of them (Patients: 1, 3, 6, 7) were also coronal imbalance preoperatively, while the other three of were coronal balance before the surgery (Fig. 1). Most obviously, all these seven patients have LIV equal to L5 level. The comparing data between final coronal imbalance and balance patients are showing in Table 2. No significant difference can be found in age, gender or Risser sign between patients with final coronal imbalance and balance. The mean lumbar curve Cobb angle, in imbalance group was 55.5° preoperatively and 4.1° at last with mean corrective rate of 93.5%, while in balance group was 52.6° preoperatively and 7.2° postoperatively with mean corrective rate of 86.4%. No significant difference was found in preoperative or postoperative Cobb angle between imbalance and balance group. When comparing preoperative LEV or LIV tilt and translation (that used to be considered as predictive parameters for postoperative coronal imbalance), we found there was no significant difference in preoperative LEV tilt (32.1° vs. 30.3°, *p* = 0.112) or translation (32.5 mm vs. 26.2 mm, *p* = 0.096) between final coronal balance and imbalance group; But both preoperative LIV tilt and translation (5.2 mm) in imbalance group were significantly lower than balance group (11.4° vs. 21.7°, *p* = 0.001 and 5.2 mm vs. 15.7 mm, *p* = 0.022, respectively). The preoperative reduce-bending lumbosacral hemi-curve was 15.3° in final imbalance group that significantly higher than 6.3° in balance group (p = 0.001).

**Table 1:** The demographic and radiographic data in all seven patients with final coronal imbalance

Case	Sex	Age	Pre-op. CB	Pre-op. ML (°)	Final ML (°)	LEV	LIV	Pre-op. LEV tilt (°)	Pre-op. LEV Trans(mm)	Pre-op. LIV(L5) tilt (°)	Pre-op. LIV(L5) Trans(mm)
1	F	16	I	46.1	−2.8	L3	L5	41.6	22.3	6.0	6.3
2	F	13	B	76.6	10.0	L3	L5	27.2	44.7	17.9	7.9
3	F	20	I	56.5	11.1	L4	L5	34.9	24.8	14.5	5.4
4	M	16	B	45.0	1.6	L4	L5	24.3	28.8	8.0	4.4
5	F	15	B	50.2	0.7	L3	L5	26.6	36.4	13.0	6.1
6	F	13	I	66.7	4.5	L3	L5	38.3	39.9	9.9	3.9
7	M	12	I	52.7	3.9	L3	L5	31.6	30.6	10.4	2.4

*Pre-op* preoperative, *CB* Coronal Balance (B means Balance while I means Imbalance), *ML* Main Lumbar Curve, *LEV* lowest end vertebra, *LIV* lowest instrument vertebra, *Trans* Translation

**Table 2:** Comparing coronal parameters between final coronal balance and imbalance Lenke 5 AIS patients (*n* = 56)

	Imbalance (*n* = 7)	Balance		P_1_	P_2_
		All (*n* = 49)	LIV = L5 (*n* = 14)		
Age (ys)	15.0 ± 2.7	15.2 ± 2.0	15.8 ± 2.5	0.829	0.531
Risser Sign (°)	2.6 ± 1.8	3.5 ± 1.3	3.9 ± 1.4	0.224	0.113
Gender				0.673	0.525
Female	5	31	8		
Male	2	18	6		
ML Cobb					
Pre-op(°)	55.5 ± 12.5	52.6 ± 7.7	55.6 ± 9.1	0.392	0.986
Last (°)	4.1 ± 5.0	7.2 ± 6.9	8.0 ± 9.1	0.249	0.301
CR (%)	93.5 ± 8.3	86.4 ± 12.2	86.5 ± 13.0	0.143	0.211
C7-CSVL (mm)					
Pre-op	19.9 ± 19.6	13.7 ± 17.5	14.0 ± 13.6	0.394	0.426
Last^a^	23.9 ± 25.5	6.1 ± 9.3	8.0 ± 7.5	0.001	0.04
Pre-op LEV Tilt (°)	32.1 ± 6.5	30.3 ± 6.2	28.6 ± 5.2	0.112	0.544
Pre-op LEV Trans (mm)	32.5 ± 8.2	26.2 ± 8.4	26.6 ± 8.1	0.096	0.134
Pre-op LIV tilt (°)	11.4 ± 4.1	21.7 ± 7.4^a^	14.2 ± 5.5	0.001	0.252
Pre-op LIV Trans (mm)	5.2 ± 1.8	15.7 ± 11.6^a^	5.0 ± 2.1	0.022	0.812
Pre-op Hemi-LS (°)					
Neutral	27.9 ± 6.1	23.8 ± 6.8	25.7 ± 8.0	0.142	0.519
Reduce^a^	15.3 ± 4.8	6.3 ± 6.3	7.5 ± 6.3	0.001	0.009
LIV (n)^a^				0.008	/
L3	0	12	/		
L4	0	23	/		
L5	7	14	14		

P1 means the *p* value of imbalance vs. All balance patients; P2 means the *p* value of imbalance vs. Balance with LIV = L5 group

*ML* main lumbar curve, *Op* operation, *Trans* translation, *Hemi-LS* lumbosacral Hemi-curve

^a^means significant different

We subsequently compared final coronal imbalance patients (all with LIV = L5) with other LIV = L5 patients who achieved normal coronal balance in the last follow-up (Balance & LIV = L5 subgroup, *n* = 14). (Table 2). We found no significant difference exist in age, gender, Risser sign, main TL/L Cobb angle, preoperative LEV tilt/translation, or preoperative LIV tilt/translation between these two subgroups. And We found that the only significant difference exist in the preoperative reduce-bending lumbosacral hemi-curve Cobb angle, which was 15.3° in imbalance group, marking higher than 7.5° in balance & LIV = L5 subgroup (*p* = 0.009).

### Sagittal balance

According to our sagittal imbalance criteria, there were 34/56 (61%) patients defined as sagittal imbalance before surgery. There were 21 female and 13 male with mean age of 15.4 years, mean Risser sign of 3.3. No significant difference was found in age, gender or Risser sign between sagittal balance and imbalance patients (Table 3). The preoperative main TL/L Cobb angle in sagittal imbalance group was 51.9 ± 8.3°, which was very similar to the angle of 54.5 ± 8.4° in sagittal balance group (*p* = 0.781). When comparing the sagittal parameters between these two groups, we also found there was no significant difference in SS, LL, TK or even SVA distance. However, the mean PI and PT value in sagittal imbalance patients was 41.3° and 1.4° respectively, both which were significantly lower than the value of 49.2° and 10.3° in patients with normal sagittal balance (Table 3).

**Table 3:** Comparing sagittal parameters between preoperative sagittal balance and imbalance Lenke 5 AIS patients (*n* = 56)

	Imbalance (*n* = 34)	Balance (*n* = 22)	*P*
Age (years)	15.4 ± 2.2	15.0 ± 2.0	0.465
Risser	3.3 ± 1.5	3.5 ± 1.3	0.524
Gender			0.625
Female	21	15	
Male	13	7	
PI (°)^a^	41.3 ± 9.2	49.2 ± 10.7	0.006
SS (°)	39.9 ± 7.0	38.9 ± 8.3	0.66
PT (°)^a^	1.4 ± 8.5	10.3 ± 4.3	0.000
LL (°)	52.5 ± 10.9	47.9 ± 9.7	0.786
PI-LL (°)^a^	−11.2 ± 13.7	1.2 ± 6.7	0.000
TK (°)	26.5 ± 15.6	25.8 ± 10.8	0.865
SVA (mm)	−14.4 ± 34.5	0.2 ± 18.6	0.078

*PI* pelvic incidence, *SS* sacral slope, *PT* pelvic tilt, *LL* lumbar lordosis, *TK* thoracic kyphosis, *SVA* sagittal vertical axis, *LIV* lower instrumented vertebra

^a^means significant different

At last follow-up after surgery, there were 18 in 56 sagittal imbalance patients. They were 10 female and 8 male with average age of 15.7 years, average Risser sign of 3. No significant difference was found between them and patients with final sagittal balance (Table 4). The preoperative and final main TL/L Cobb angle in them was 52.5 ± 8.5° and 8.1 ± 8.9° respectively with average corrective rate of 85.9%. The preoperative and final main TL/L Cobb angle in patients with final normal sagittal balance was 53.2 ± 8.4° and 6.2 ± 8.5° with mean corrective rate of 88.1%. However, there was no significant difference in either MAIN TL/L Cobb angle or corrective rate. When comparing the sagittal parameters between final sagittal imbalance and balance patients, the SS, LL, TK and SVA results were similar (Table 4). But the final sagittal imbalance group had significantly lower PI, PT and PI-LL value than balance group.

**Table 4:** Comparing sagittal parameters between final sagittal balance and imbalance Lenke 5 AIS patients (*n* = 56)

	Imbalance (*n* = 18)	Balance (*n* = 38)	*P*
Age	15.7 ± 2.3	14.9 ± 2.0	0.203
Risser	3.4 ± 1.6	3.4 ± 1.3	0.861
Gender			0.348
Female	10	26	
Male	8	12	
PI (°)^a^	37.7 ± 9.4	48.0 ± 9.5	0.000
SS (°)	34.1 ± 7.2	36.7 ± 7.4	0.23
PT (°)^a^	3.5 ± 9.3	11.3 ± 6.1	0.001
LL(°)	50.4 ± 8.6	51.1 ± 8.5	0.764
PI-LL(°)^a^	−12.7 ± 12.6	−3.1 ± 7.0	0.001
TK(°)	31.9 ± 14.1	30.5 ± 11.8	0.694
SVA (mm)	−15.3 ± 29.2	−10.0 ± 21.7	0.465
LIV (level)			0.259
L3	2	10	
L4	8	15	
L5	8	13	

*PI* pelvic incidence, *PT* pelvic tilt, *LL* lumbar lordosis, *TK* thoracic kyphosis, *SVA* sagittal vertical axis, *LIV* lower instrumented vertebra

^a^means significant different means significant different

**Fig. 2:**
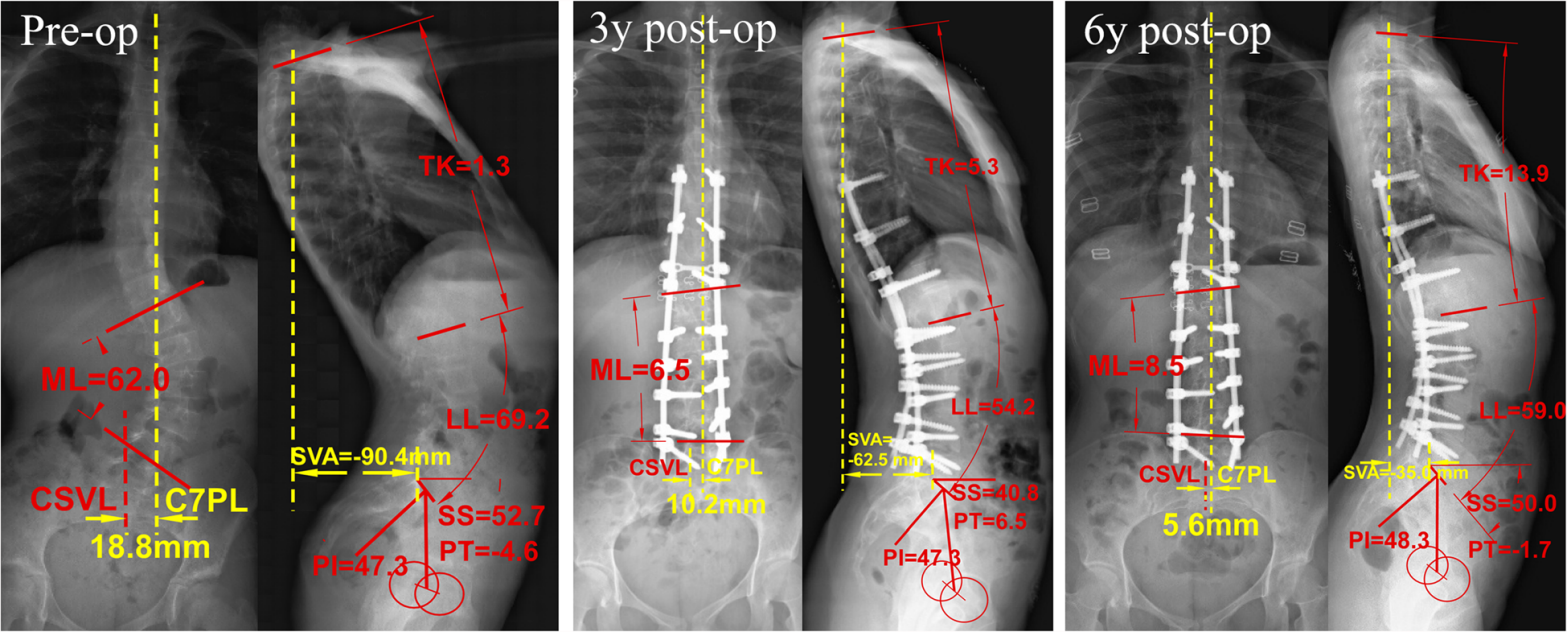
The pre-, 3-year post- and 6-year post-operative X rays of a Lenke 5 AIS patient with initial and final sagittal imbalance. This patient had main lumbar Cobb angle of 62.0° before surgery and 8.5° at 6 years after surgery with a corrective rate of 86.3%. And coronal balance was always normal. However, she had severe sagittal imbalance preoperatively (SVA = − 90.4 mm, PT = − 4.6° and PI-LL = − 21.9°). At 3 years after surgery, thought the pelvic posture has restored to normal (PT = 6.5°) with a normal PI-LL matching (PI-LL = − 6.9°), however her sagittal imbalance was still exist with a SVA = − 63.5 mm. At final follow-up (6 years), the sagittal imbalance has still not compensated to normal. Fused to L5 has sacrificed most of the sagittal compensatory ability and this should be an important reason for her final sagittal imbalance

When analyzing the predictive factor of final sagittal imbalance, we found LIV selection was another remarkable factor beside PI. In LIV = L3 group, there were 8 in total 12 patients showed sagittal imbalance before surgery, while 6 of them recovered to normal sagittal balance from surgery. In LIV = L4 group (*n* = 23), there were 17 patients showed sagittal imbalance preoperatively. Among them, 10 out of the 17 patients who initially suffered from sagittal imbalance recovered to normal balance after surgery, while there was only 1 patient whose initially normal sagittal balance deteriorated into imbalance after surgery. Overall 8 patients showed sagittal imbalance at the last follow-up. In LIV = L5 group (*n* = 21), there were 9 sagittal imbalance patients initially while still 8 imbalance patients at last follow (Fig. 2). Only 3 in 9 patients with abnormal sagittal balance get improve to normal from surgery, meanwhile 2 initial normal patients get worse to sagittal imbalance after surgery. The sagittal balance recover rate in different groups was 75% in LIV = L3 (the highest), 58.8% in LIV = L4, and 30% in LIV = L5 group (the lowest).

### Coronal + sagittal balance

The correlation analysis was done between the patients who suffered from preoperative coronal imbalance and the one who suffered from preoperative sagittal imbalance patients, but no significant correlation showed (*r* = 0.024, *p* = 0.865). Also, no marked correlation can be found between the patients with final coronal imbalance and the ones with final sagittal imbalance (*r* = 0.191, *p* = 0.171). From “Coronal Balance” and “Sagittal Balance” results section presented above, we found that selecting LIV on L5 should be a high risk factor for both coronal imbalance and sagittal imbalance at last follow-up. In all 21 patients with LIV = L5, however, the correlation analysis showed that there was still no direct correlation between the patients with final coronal imbalance and the ones with final sagittal imbalance (*r* = 0.277, *p* = 0.224).

## Discussion

The global spinal balance of Lenke 5 AIS patients is a critical evaluation indictor for the operative effects in these patients. Until now, a number of previous studies focused on the coronal balance only in these patients, while few discussed the sagittal balance only and still none has analyzed the coronal and sagittal balance at the same time.

Coronal balance has long been an important evaluation indicator for the surgical outcomes in patients with AIS [13], and using C7-CSVL distance >20 mm as the criteria for judging the coronal imbalance is already widely accepted. In the patients with Lenke 5 AIS, few previous studies have noticed the selection of LIV should be significantly associated with postoperative coronal imbalance [2, 4, 5]. Li et al. [5] have initially analyzed four of the twenty-seven Lenke 5 AIS patients who demonstrated global coronal imbalance immediately after posterior surgery, and found their common radiographic feature was preoperative LIV tilt≧25° and failed to reduce below 8° postoperatively. Then they advocated extending the fixation to one more distal level when the planning LIV tilt exceeds 25°. Subsequently, Wang et al. [2] supported and extended above argument and then suggested a translation of 28 mm and a tilt of 25° might be used as the general criterion for selecting LIV. However, their conclusion was drawn from a meta-analysis and a pure mathematics linear regression formula between pre- and post- operative balance distance parameter. But actually, only 4 in 30 their patients performed real coronal imbalance (C7-CSVL distance > 20 mm) in their follow-up.

Before long, an opposite voice was found in another study by Sun et al. [8]. They found that none of their patients who developed coronal imbalance at their last follow-up performance above radiographic characteristics, and that the other two patients who had LIV tilt > 25° preoperatively and failed to reduce below 8° postoperatively did not suffer from coronal imbalance in the end. In our present study, we found no patients with final coronal imbalance had a preoperative LIV tilt exceeding 18° (Table 1 and Fig. 1). The mean preoperative LIV tilt in coronal imbalance patients was 11.4° which was significantly lower than the mean preoperative LIV tilt of 21.7° in coronal balance ones. Furthermore, the LIV tilt exceeding 25° performed in 20 of 53 patients preoperatively and failure to reduce below 8° were found in 3 of them postoperatively, but none of these showed coronal imbalance at the last follow-up. So, it indicated that there are other factors playing a more important role than LIV tilt or translation in giving rise to a final coronal imbalance.

In fact, very few Lenke 5 patients that performed postoperative coronal imbalance have been reported in previous studies, especially in those with follow-up longer than 2 years [2, 4, 5, 8]. In our study, we found 23 out of 56 patients showed coronal imbalance before surgery, but only 7 patients still remained their poor coronal balance until the final follow-up (24 to 72 months). And all these 7 patients were our early-period cases that with a longer fusion to L5. It shows that postoperative coronal imbalance should be a rare complication following posterior surgery if we can stop the fusion above or at least equal to L4. If we kept more than two mobilizable levels below the fused section, almost all patients’ coronal trunk could be self-adjusted to balance given a sufficiently long enough follow-up period. Even for those patients who already had coronal imbalance before surgery or who been observed coronal imbalance at the early follow-up stage, according to this principle, no patient still keeps imbalance to the end in present study. Conversely, if we fused on L5, then there was a high risk of postoperative coronal imbalance.

Compared to preoperative translation or tilt of the lowest vertebrae that are planned to be fused, it seems the flexibility of lumbosacral hemi-curve is more important for predicting final coronal imbalance. In order to obtain more deformity correction and better coronal balance in Lenke 5 patients, Shufflebarger et al. suggested it’s best to choose the LEV as the LIV if the LEV can turn to horizontal on the side-bending films [14]. In a more recent retrospective study by Yang et al., they found only L5 tilt on the bending radiographs show strong correlation with postoperative coronal imbalance [6]. Our present results are similar to above both. When comparing the coronal balance patients and imbalance ones those with the same LIV, we find the only significant parameter is the preoperative reduce-side bending LEV-S1 Cobb angle, which shows higher in the imbalance subgroup with a critical point of 15° (value greater means risk higher). This angle on one side represent the flexibility of lumbosacral hemi-curve, on the other side predict the compensatory ability of the motion segment still be kept after fusion surgery. And it should be an important risk factor of final coronal imbalance in Lenke 5 patients, particularly for the ones necessary to be fixed to L5. In other words, for a case with preoperative reduce-side bending LEV-S1 Cobb angle greater than 15°, it is necessary to stop fusion above (or at most equal to) L4 for achieving better postoperative coronal balance.

Due to our advancing understanding of the fact that scoliosis is a kind of three-dimensional deformity, more recent studies start to be concerned about sagittal alignment improvement as another important surgical outcome besides the coronal plane correction [3, 15–18]. Moreover, sagittal balance has been demonstrated as a much stronger predictor of improved functional outcomes than coronal balance in adult patients with spinal deformity [15]. Although insufficient evidences exist at present directly indicating this predictive effect of sagittal balance in adolescent idiopathic scoliosis patients, nevertheless, theoretically, the effect should be significant to these young patients given the fact of their future growth and degeneration.

It is still a debate on the sagittal balance evaluation criterion. In the past, sagittal imbalance has been defined relying on the distance parameter between C7 and sacrum posterior superior corner (SVA), which was similar to coronal imbalance criterion. But today, this concept is being updated. There is now a consensus that sagittal balance should be understood as an interaction of the spine and pelvis which adjusting body gravity center above the hip joint and keeping standing posture. In addition to SVA, pelvic tilt (PT) and mismatch between pelvic incidence and lumbar lordosis (PI-LL) are proved to have equally important role in sagittal balance [19, 20]. So, in present study, our sagittal imbalance criteria draw from the synthesis of our previous research results in Lenke 5 AIS and the SRS-Schwab classification theory in adult spine deformity [3, 12]. And sagittal imbalance was identified when one or more of the following items exist: 1. SVA > 40 mm; 2. PT < PI/10 or PT > 20°; 3. PI-LL > 10°.

We found 61% (34/56) our present patients show sagittal imbalance preoperatively, and 32% (18/56) ones show imbalance at the last follow-up. The anteverted pelvis, which has first been put forward by our previous study [3], was now found to be the most common sagittal imbalance pattern in the patient with Lenke 5 AIS. Preoperatively the PI value in sagittal imbalance group was significantly lower than balance group (41.3° vs. 49.2°). Moreover at last follow-up, the mean PI value in patients with final sagittal imbalance was 37.7° which was the lowest one. It suggested the lower PI value should be one of the reasons for sagittal imbalance in Lenke 5 patients. Idiopathic scoliosis occurring in thoracolumbar or lumbar spine always axially rotates these vertebrae and then reduces segmental lordosis (usually upper lumbar) [21, 22]. The lower lordosis increases subsequently, and accompanied by anterior tilt of pelvis as a compensatory. Smaller PI generally represents lower pelvic sagittal adjustment ability [23]. So, those Lenke 5 AIS patients with a smaller PI are easier to reach their pelvic compensatory limit and show an unbalanced anteverted position. And that for the patients with PI lower than 39°, there is a high risk of long-term uncorrected sagittal imbalance posture after posterior surgery [3].

Besides individual PI value, the choice of LIV should be another important factor related to the postoperative sagittal imbalance. We found there was a gradient difference in postoperative sagittal imbalance improvement among patients with LIV equal to L3, L4 or L5. At the final follow-up, the patients with LIV selected in L5 performed the worst sagittal balance in all three groups; while patients in LIV equal to L3 group showed slightly better sagittal balance than L4 group. The regulation of sagittal alignment following posterior fusion mainly comes from two aspects: the lumbar lordosis within fusion level and the lordosis below fusion [24]. The former one depends on the rods’ sagittal curvature bended during operation. Thought ideally the rod curvature should be designed and bended according to individual PI value, however, it usually very difficult to be controlled accurately in practice. And most times, for these AIS patients, our attention is more likely to focus on the correction of coronal curve, rather than sagittal. So, it suggests in fact the lumbar lordosis compensation below fusion level plays more important role in the sagittal balance adjustment. When distal fixation extending to L5, the compensative capacity of the only mobilizable segment remained is very limited. In this case, the imbalance sagittal that generally existed in Lenke 5 AIS patients will be hard to correct through the surgery (Fig. 2).

When comprehensively taking coronal and sagittal plane into account, though no significant correlation showed between the coronal and sagittal balance based on statistical results, from the above analysis it can be inferred that the LIV selection plays an important role in both the coronal and sagittal balance respectively. In making the preoperative plan, it should be noted that fusing to L5 will bring a high risk of postoperative coronal imbalance or/and sagittal imbalance.

For the Lenke 5 AIS patients with severe curve, previous studies recommended extending posterior fusion distal level to LEV+ 1 for better scoliosis correction [8]. So sometimes there is a need to fuse on L5. However, our study in the opposite adds to the evidence that L5 is the secondary choice in this case. When L5 fusion is inevitable, close attention should be paid to some important predictive parameters, such as reducing LEV-S1 Cobb in the coronal plane and PI in the sagittal plane, so as to obtain an ideal trunk coronal and sagittal balance. Every attempt should be made to avoid sacrificing lumbar motion segments so as to obtain more coronal correction of scoliosis.

A number of limitations exist in this study. Firstly, there was relatively small number of patients in present study. Especially with regard to analyze the coronal imbalance, the current sample size (only 7 final imbalance patients) in fact is so small to draw a significant and accurate conclusion. In response to this, the coronal imbalance influencing factors we obtained in this study tend to be a hypothesis, which should be further confirmed by large sample size in future. Secondly, only radiographic parameters of coronal or sagittal balance have been compared here and no patient’s functional outcomes or satisfaction included. This should be taken into account in future studies so as to find out whether the trunk imbalance, especially the sagittal imbalance, leads to a poor result in the health-related quality of life in these AIS patients.

## Conclusion

Both coronal imbalance and sagittal imbalance are common symptoms found in Lenke 5 AIS patients. With the posterior correction and fusion, it will be easier to correct the imbalance in coronal plane to balance than the imbalance in sagittal plane. And whether these patients will still suffer from postoperative coronal and sagittal imbalance is determined by the selection of LIV. Moreover, for the patients with LIV in L5, the stakes of suffering from trunk imbalance (coronal imbalance or sagittal imbalance) are spectacularly high. It is more important for these patients to save more motion segments (at least 2 levels) below fusion area than gain more scoliosis correction. Apart from LIV, the reduce-side bending LEV-S1 Cobb angle is another predictive factor for postoperative coronal imbalance: when the angle is greater than 15° before surgery, a patient is more likely to suffer from postoperative coronal imbalance. And in sagittal plane, PI is another important predictive factor for postoperative sagittal imbalance besides LIV: the patient with a lower PI value than 39° is more likely to suffer from postoperative sagittal imbalance after surgery. For the cases who need to be fused to L5, the two parameters (reduce-side bending LEV-S1 Cobb angle and PI) should be taken into account before surgery.

## Abbreviations

AIS: Adolescent idiopathic scoliosis
LEV: Lower end vertebra
LIV: Lower instrumented vertebra
LL: Lumbar lordosis
PI: Pelvic incidence
PT: Pelvic tilt
SS: Sacral slope
SVA: Sagittal vertical axis
TK: Thoracic kyphosis
TL/L: Thoracolumbar or lumbar
UIV: Upper instrumented vertebra
